# Identification of multimorbidity hub genes for knee osteoarthritis-atherosclerosis and potential clinical applications

**DOI:** 10.3892/mmr.2025.13750

**Published:** 2025-11-12

**Authors:** Qingyuan Kang, Ping Yuan, Peisen Xie, Wentao Xiao, Liguang Dong, Zhenpeng Guan, Keshi Zhang

**Affiliations:** 1 School of Basic Medicine, Shenyang Medical College, Shenyang, Liaoning 110034, P.R. China; 2 Department of Orthopedics, Peking University Shougang Hospital, Beijing 100144, P.R. China; 3 Technology Branch, Aerospace Medical and Health Science and Technology Group Co., Beijing 100089, P.R. China; 4 Second School of Clinical Medicine, Xinjiang Medical University, Ürümqi, Xinjiang Uyghur Autonomous Region 830054, P.R. China; 5 Medical Examination Department, Peking University Shougang Hospital, Beijing 100144, P.R. China

**Keywords:** knee osteoarthritis, atherosclerosis, multimorbidity, bioinformatics, caffeine, hub genes

## Abstract

The combination of knee osteoarthritis (KOA) and atherosclerosis (AS) is a common multimorbidity. Epidemiological studies have demonstrated the existence of common risk factors, with metabolic syndrome possibly considered the most critical. In the present study, metabolism-related clinical information was analyzed and metabolic profiles were assessed in healthy controls, patients with KOA, patients with AS and patients with both conditions using untargeted serum metabolomics assays. Potential KOA-AS multimorbidity hub genes were identified using transcriptomics datasets from the Gene Expression Omnibus database and were validated using clinical samples and animal experiments. Finally, the clinical applications of the analyzed biomolecules were predicted. The results showed that the caffeine metabolic pathway was markedly associated with KOA-AS multimorbidity and caffeine interacted with two potential hub genes (*EGR1* and *GSK3B*). In the validation experiment using clinical samples, early growth response 1 (Egr1) protein was only associated with AS. In the mouse disease model, Egr1 protein in the serum and cartilage was associated with KOA-AS multimorbidity, with consistent expression trends. Receiver operating characteristic (ROC) analysis showed three metabolites with an area under the ROC curve of >0.7; drug prediction yielded two drugs that interacted with *EGR1.* In conclusion, KOA-AS multimorbidity may be associated with metabolic abnormalities in the early stages and could develop into chronic inflammation in the later stages. Through multi-omics analysis, three caffeine-related metabolites with diagnostic value were obtained and *EGR1* was identified as the key gene for KOA-AS multimorbidity.

## Introduction

As average life expectancy increases and the population ages, the global disease spectrum shifts (1). Chronic disease multimorbidity has become common (2), with multimorbidity rates as high as 55–98% in the elderly population (3). Notably, patients with multimorbidities tend to be in poorer health, with higher rates of mortality and disability, leading to an increased consumption of healthcare resources (4). Osteoarthritis (OA) and cardiovascular diseases (CVDs) are common chronic degenerative diseases and their combination is a frequent type of multimorbidity (5). The two diseases are the leading causes of disability and death in individuals aged >65 years, respectively (6–8) and their progressively higher incidence and increasingly younger onset cause a heavy burden of disease (9–11), posing a notable threat to health.

Knee OA (KOA) is the most common type of OA and >50% of patients with symptomatic KOA experience severe disability with limited activities of daily living (12). Atherosclerosis (AS), as the most important etiological and pathological basis of CVDs (13), contributes to ~45% of CVD-associated mortalities and >20 million individuals worldwide succumb to AS each year (14). A large number of existing epidemiological studies have confirmed the association between OA and CVDs (15). Individuals with OA have a higher risk of developing and dying from CVDs (16–19) and the severity of OA is associated with CVD mortality. Furthermore, individuals at high risk of CVDs have a higher probability of developing OA (20) and high levels of AS markers are associated with a high prevalence of imaging KOA (21,22). Previous studies have also shown that popliteal artery wall thickness is positively associated with tibial cartilage loss (23,24). This evidence indicates that OA and CVDs may be caused by common risk factors, or that other complex and as yet unappreciated associations between them exist.

Using KOA and AS as an entry point, the present study aimed to explore the link between KOA and AS to enrich the research base of OA-CVD multimorbidities. For the two main disease subtypes under consideration, KOA and AS share common risk factors, such as age, obesity, hypertension, diabetes mellitus and abnormal lipid metabolism (25). A previous study suggested that metabolic syndrome mediates the development of KOA-AS multimorbidity (16), but confirmatory studies are lacking. Multi-omics technology is a research method that can be used to carry out a more comprehensive exploration by integrating data from different histological levels, thus fully revealing the characteristics of disease. In order to determine whether metabolic abnormalities serve a key role in KOA-AS multimorbidity and to identify which key biomolecules are associated with KOA-AS multimorbidity, differences in metabolism-related clinical indices among different diseased populations were analyzed. In addition, possible evidence was explored at the molecular level through multi-omics analysis, which may improve the understanding of the relationship between KOA and AS, further promote the prevention and treatment of KOA-AS and improve future multimorbidity prevention and disease management.

## Materials and methods

### Study population

After excluding individuals unable to undergo radiological examination or those with severe underlying diseases, 172 subjects from Peking University Shougang Hospital (age, 20–60 years, mean age: 40 years, 71.5% male) undergoing physical examinations volunteered for additional KOA and AS screening, were recruited to the present study from July to August 2024. The subjects were offered additional screening after signing an informed consent form. Based on the examination results, the subjects were divided into the following four groups: The healthy control group (HC), the KOA group, the AS group and the KOA-AS multimorbidity group (MM). The HC group consisted of healthy individuals without KOA and AS; KOA group consisted of patients with KOA without AS; the AS group consisted of patients with AS without KOA; and the MM group consisted of patients with both KOA and AS.

### Disease screening methods

KOA was diagnosed using American College of Rheumatology criteria (26). AS was diagnosed using brachial-ankle pulse wave velocity (baPWV), with patients considered to have AS when baPWV was ≥2 times SD.

### Patient information

The clinical data of the subjects were derived from the laboratory information system and mainly included: i) Basic information, such as sex and age; ii) general examination of height, weight and body mass index (BMI); iii) laboratory indicators, including lipids (triglycerides, total cholesterol), fasting blood glucose and blood pressure (systolic, diastolic); and iv) a history of previous diseases.

### Metabolomics assays

The test samples were derived from residual serum samples obtained from the aforementioned patient population. The serum samples were extracted with 80% methanol in water, vortexed and shaken and then maintained in an ice bath for 5 min. The samples were then centrifuged at 15,000 × g for 20 min at 4°C and a certain amount of supernatant was diluted with mass spectrometry (MS)-grade water until the methanol content was 53%. The samples were again centrifuged at 15,000 × g for 20 min at 4°C, the supernatant was collected and the samples underwent liquid chromatography (LC)-MS for analysis.

Chromatographic separation was performed using a Hypersil GOLD column (C18) and an ACQUITY UPLC BEH Amide Column (100×2.1 mm, 1.9 µm; Thermo Fisher Scientific, Inc.). Metabolites eluted from the column were detected using a Q Exactive HF/Q Exactive HF-X super-resolution liquid-mass spectrometer (Thermo Fisher Scientific, Inc.). MS often uses two scanning modes, positive ion scanning and negative ion scanning, for data acquisition during the detection process. In addition, to assess the stability of the LC-MS throughout the acquisition process, eight quality control samples were randomly distributed in a pool of all samples.

### Metabolomics analysis

The downstream data files were converted to mzXML format using ProteoWizard software (version 3.0) (27) and then XCMS software (version 2006) (28) was used for peak extraction and peak quantification. Peak alignment was performed and peak area correction was performed with the first quality control (QC) sample. Metabolite identification was then performed based on setting 10 ppm mass deviation and information such as addition ions against a high-quality secondary spectral database (novogene.cn/). Subsequently, the background ions were removed with blank sample measurements and the raw quantitative results of each sample to be tested were normalized. Finally, compounds with a coefficient of variation (CV) of the relative peak area of >30% in the QC samples were deleted (29) and the number of metabolites identified and relative quantitative value results were obtained. The data processing was partially performed based on a Linux operating system (CentOS Version 6.6; linux.org/) and R package (version 3.4.3, rstudio.com/) and Python software (version 3.5.0, http://www.python.org/).

The metabolomics data processing software metaX (version 2017) (30) was used to transform the data and partial least squares discriminant analysis (PLS-DA) was performed to obtain the variable importance in projection (VIP) values of the metabolites. Univariate analysis was based on paired t-test to calculate the P-value between the groups for each metabolite and the multiplicity of difference [fold change (FC) value] between groups for metabolites. The screening criteria for differential metabolites were VIP>1, P<0.05 and FC≥2 or FC≤0.5. Volcano plots were plotted using the R package ggplot2 (R-3.4.3) and clustered heat maps (P<0.05 being statistically significant; t-test) and the Pearson correlation coefficients heat maps (r-values≥0.6 indicating moderate strength correlation) were generated using the pheatmap package (R-3.4.3). In addition, correlation maps were plotted using the corrplot package (R-3.4.3)in R.

The Kyoto Encyclopedia of Genes and Genomes (KEGG) database (https://www.genome.jp/kegg/pathway.html) was used to analyze the function of metabolites and the associated pathways, which were considered to be enriched when x/n>y/N; the pathways were considered to be markedly enriched when the P-value of the pathway was <0.05 and the bubble plots were visualized using the R package ggplot2.

### Transcriptomics data source

The Gene Expression Omnibus (GEO) database (ncbi.nlm.nih.gov/geo) is a gene expression database created by the NCBI that contains microarray datasets and high-throughput sequencing data. To understand the changes in gene expression that may be caused by metabolism, transcriptomics datasets with ~60 years (average age) were searched for. The key words ‘Osteoarthritis’ and ‘Atherosclerosis’ were used to search and screen the gene expression datasets at the mRNA level for analyzing the hub genes of KOA-AS multimorbidity. The inclusion and exclusion criteria for dataset selection were as follows: i) Test specimens should be *Homo sapiens* with consistent specimen types; ii) gene expression profiles should include both case and control groups; iii) experimental data expression types (sequencing platforms) should be consistent; iv) preference should be given to datasets with large sample sizes; and v) patients receiving clinical interventions should be excluded.

### Transcriptomics analysis

Differentially expressed genes (DEGs) were identified using the Limma package of the online analysis platform GEO2R (https://www.ncbi.nlm.nih.gov/geo/geo2r/). Based on the Bayesian test, the criteria for determining the DEGs of the two profiles were set as adjusted P-value <0.05 (Benjamini-Hochberg method) (31) and |logFC|>0. The common DEGs were obtained from the Venn diagrams drawn by the online mapping tool ImageGP (bic.ac.cn/BIC/#/).

The KEGG database was used to analyze the function of the hub genes and the related biological processes. When x/n>y/N, the process was considered enriched; when the P-value of the biological process was <0.05, it was considered markedly enriched and the bubble plots were visualized with the R package ggplot2.

The DEGs common to OA and AS were imported into the STRING (http://www.string-db.org/) database and a composite score of ≥0.4 was selected for protein-protein interaction (PPI) network construction, excluding proteins with no interactions and retaining only proteins in the interaction network and the results were exported in ‘tsv’ format. The results were then imported into Cytoscape (32) and the cytoHubba (https://cytoscape.org/) plug-in was used to assign a value to each protein according to the topological network algorithm and the hub genes corresponding to the key proteins were sorted and screened (33).

### Metabolite-gene network analysis

MetaboAnalystR 6.0 (34) was used to analyze the interactions between core metabolites and hub genes. The ‘Network Analysis’ module was selected online and the ‘Official gene symbol’ of the hub gene to be analyzed was imported into the database together with the ‘Compound Name’ of the core metabolite. Subsequently, the metabolite-gene interaction network was constructed by selecting the species to identify possible interactions.

### Mice and disease models

Disease modeling was performed using 6–8-week-old male mice (weight, 20–22 g). Wild-type C57BL/6 mice and APOE^−/−^mice were purchased from SPF (Beijing) Biotechnology Co., Ltd. A total of six C57 mice were used as healthy controls and 18 APOE^−/−^mice were used to construct the disease model. Mice were individually housed under standard lighting conditions (12-h dark/light cycle) and constant temperature (22±2°C) with free access to standard food and water. The animal experiments was approved by the Ethics Committee for Experimental Animals of Beijing JinglaiHuake Biotechnology Co (approval no. JLHK-20241110-02, approved date November 13, 2024). During the surgery, experimental animals were anesthetized via inhalation of isoflurane (5% induction and 2% maintenance) and the mice were euthanized by cervical dislocation at the time of tissue sampling. APOE^−/−^ mice were randomly grouped to construct AS, KOA and KOA-AS multimorbidity models. The AS disease model was constructed using the high-fat chow-feeding method with high-fat feeding for 12 weeks; the KOA disease model was surgically constructed using the modified Hulth method (35); and the KOA-AS multimorbidity model was constructed using the two techniques. C57 mice used as healthy controls were fed normal feed without treatment.

### X-ray examination

To assess the effects of KOA disease modeling, radiography was used to assess joint degeneration. To validate the construction of the KOA disease model, changes such as narrowing of the joint space and the presence of osteophytes should be observed.

### Oil Red O staining

The aortic Oil Red O staining method was used to determine the status of the AS disease model. The intact aortic tissues were fixed overnight in 4% paraformaldehyde at 4°C. All subsequent steps were performed at room temperature. The fixed tissues were rinsed with PBS and then immersed in 60% isopropanol for 30 sec. Next, the tissues were stained with Oil Red O working solution for 30 min, followed by differentiation in 60% isopropanol for 30 sec. Finally, the tissues were rinsed again with PBS and imaged on a white background. If notable red atherosclerotic plaques were detected after staining, the AS disease model was considered successfully constructed.

### ELISA

Concentrations of Egr1 and GSK3β in patient serum were determined using the Human Egr1 (cat. no. EH0892; Wuhan Fine Biotech Co., Ltd.) and the Human GSK3β ELISA Kit (cat. no. EH0630; Wuhan Fine Biotech Co., Ltd.), respectively, according to the manufacturer's instructions.

The concentrations of Egr1 and GSK3β in mouse serum were determined using the Mouse Egr1 ELISA Kit (cat. no. EM0998; Wuhan Fine Biotech Co., Ltd.) and the Mouse GSK3β ELISA Kit (cat. no. RXW202270M6; Quanzhou Ruixin Biotechnology Co., Ltd.), respectively, according to the manufacturer's instructions.

The standard curve was fitted and the relative concentration of the target protein in the samples was obtained using CurveExpert 1.4 (curveexpert.net/).

### Reverse transcription-quantitative PCR (RT-qPCR)

RNA was isolated from mouse cartilage tissue using TRIzol^®^ (Invitrogen; Thermo Fisher Scientific, Inc.). First-strand cDNA was synthesized using a RT kit (Shanghai Yeasen Biotechnology Co., Ltd.; cat. no. 11141ES60) according to the manufacturer's instructions. qPCR was performed on a Molarray MA-6000 real-time fluorescence qPCR instrument. qPCR was performed using the Realtime PCR Fluorescence Quantitative kit (Shanghai Yeasen Biotechnology Co., Ltd.; cat. no. 11201ES08) on a Molarray MA-6000 real-time fluorescence qPCR system. Thermocycling conditions were as follows: initial denaturation at 95°C for 5 min; 40 cycles of denaturation at 95°C for 10 sec, annealing at 60°C for 20 sec, and extension at 72°C for 20 sec, followed by a melt curve stage under instrument default settings. Each sample was run in three technical replicates. Relative mRNA levels were calculated with *GAPDH* as an internal reference, using the 2^−ΔΔCq^ equation (36). Primer sequences are shown in Table SI.

### Western blotting

Mouse cartilage tissue was lysed with RIPA buffer (Beyotime Institute of Biotechnology) and total protein was collected. The protein concentration was determined using a BCA assay kit. A total of 30 µg protein/lane was separated by SDS-PAGE on 12% gels and were then electrotransferred to a PVDF membrane (Merck KGaA). After blocking the membranes with protein blotting closure buffer (Biosharp Life Sciences) for 2 h at room temperature, the membranes were incubated with primary antibodies targeting Egr1 (1:1,000; cat. no. AF0589; Affinity Biosciences), GSK3β (1:5,000; cat. no. BF0695; Affinity Biosciences) and GAPDH (1:50,000; cat. no. 10494-1-AP; Proteintech Group, Inc.) at 4°C overnight. After washing three times with TBS-Tween, the membranes were incubated with HRP-labeled anti-mouse/rabbit IgG secondary antibodies (1:10,000; cat. no. bs-0295M-HRP; BIOSS) at room temperature for 1 h. The blots were then visualized using an enhanced chemiluminescence reagent (Beijing Fluorescence Biotechnology Co. Ltd.). Protein band densitometry was analyzed using ImageJ software (version 1.53e; National Institutes of Health, USA).

### Analysis of receiver operating characteristic (ROC) curves

To assess the sensitivity and specificity of metabolites as potential diagnostic markers, a ROC curve analysis was performed based on the relative quantitative values of metabolites in the HC and MM groups of the metabolomics assay using R programming software (version 3.4.3, rstudio.com/). The confidence interval was set at 95% and metabolites with an area under the ROC curve (AUC) of >0.7 were considered significant.

### Drug prediction

Small molecule targeted drugs were predicted using the Drug-Gene Interaction Database (DGidb; http://dgidb.org/), a publicly accessible resource that aggregates gene or gene product, drug and drug-drug-gene interaction records, which allows for drug prediction through genes. In turn, this drives clinical hypothesis generation and discovery by physicians and researchers (37).

### Statistical analysis

Data analysis for the omics portion of the research methodology was mainly performed using dedicated histological analysis tools/software or the R software package (version 4.2.2, rstudio.com/). Data in the epidemiological analysis and validation experiment were analyzed using SPSS version 25 statistical software (IBM Corp.), with independent samples t-test used for continuous variables and χ^2^ test for categorical variables. For comparisons across more than two groups, one-way ANOVA followed by Dunnett's post hoc test was applied. P<0.05 was considered to indicate a statistically significant difference.

## Results

### Clinical indicators analysis

To determine whether KOA-AS multimorbidity was associated with metabolic abnormalities, healthy controls and three patient groups were screened based on the diagnosis of KOA-AS (Table SII). The between-group differences regarding metabolic syndrome-related indices (BMI, lipids, blood glucose and blood pressure), as well as medical history, were comparatively analyzed (Table I).

Among the results, BMI showed differences in the KOA, AS and MM group compared with that in the HC group. Triglycerides showed differences only in the comparison between the AS and the HC group. In general, both triglycerides and total cholesterol showed an increasing trend in those who had AS (AS and MM groups) compared with those who did not have AS (HC and KOA groups). Fasting blood glucose showed differences in all five group comparisons, suggesting that abnormalities in glucose metabolism may be associated with both KOA and AS and that this could have a contributory effect on the development of KOA-AS multimorbidity. Among all metabolic abnormalities, glucose metabolism was the most strongly associated with KOA-AS multimorbidity. Systolic blood pressure (mmHg) and diastolic blood pressure (mmHg) showed differences in comparisons with the AS, the MM and the HC group and in comparisons of the MM and the KOA group, indicating that hypertension may be strongly associated with AS. Notably, hypertension is a recognized risk factor for AS.

In the analysis of past medical history, a past history of hypertension and a past history of diabetes mellitus were associated with KOA-AS multimorbidity. In general, the more history of past diseases an individual had (all diseases), the greater the probability of developing KOA-AS multimorbidity, suggesting that this multimorbidity may be associated with a variety of adverse health conditions.

Overall, in the analysis of metabolism-related clinical information, BMI, fasting glucose, a previous history of hypertension and a previous history of diabetes mellitus were associated with KOA-AS multimorbidity. By contrast, the laboratory measures of blood pressure and dyslipidemia were only associated with AS. Furthermore, at the same age, there was more evidence of metabolic abnormalities in the AS group compared with in the KOA group.

### Qualitative and quantitative metabolite analysis

To understand the association between metabolism and KOA-AS multimorbidity, the study subjects were screened, retaining redundant serum samples (residual blood from laboratory tests) among those analyzed in the previous step, the number of sample cases in the remaining subgroups was adjusted based on the subgroups with the lowest number of samples according to the ratio of 1:1 and the ratio of age and sex in each group was balanced as much as possible (Table SIII). Non-targeted serum metabolomics based on high-resolution MS was performed and the molecular peaks were matched and identified using a high-quality secondary spectral information database to reflect the differences in the characteristics of serum total metabolites in different diseased populations.

**Figure 1.**
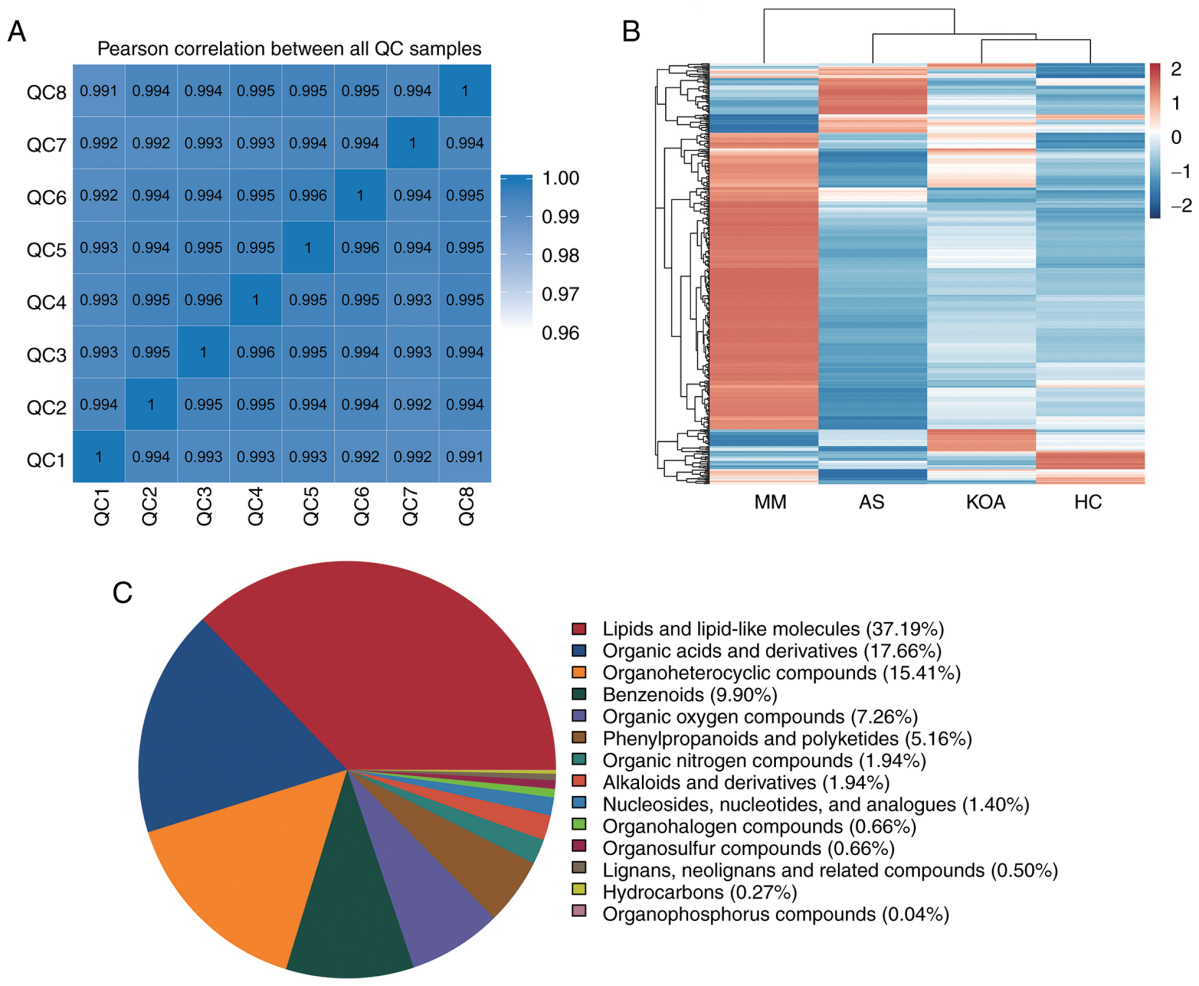
Metabolomics data quality assessment and metabolite characterization. (A) Heat map of Pearson correlation coefficient for QC samples. (B) Heatmap of metabolite clustering for between-group differences. (C) Pie chart of the distribution of detected metabolite classes in QC samples. QC, Quality Control.

Pearson correlation coefficients were first calculated between the QC samples based on the relative quantitative values of the metabolites and it was observed that the correlation coefficients (r-values) of the different QC samples were all >0.99, which indicated that the instrument was well stabilized throughout the entire sample testing period (Fig. 1A). The raw data from the downstream machine were preprocessed using XCMS software and metabolites with a CV of <30% in the QC samples were retained, resulting in a total of 2,595 identified metabolites. The top five metabolite classes were: Lipid and lipid-like molecules, organic acids and their derivatives, organic heterocyclic compounds, benzenes and organic oxygenated compounds (Fig. 1C).

### Intergroup cluster analysis of all differential metabolites

In order to visualize the overall changes in serum metabolism, hierarchical clustering analysis (38) of all differential metabolites between the groups was performed. The results showed that the metabolic profiles of the KOA group were the most similar to those of the HC group, followed by the AS group and lastly the MM group. This is consistent with the results of the present analysis of clinical indicators, the metabolic abnormalities were more significant in the AS group compared with those in the KOA group (Fig. 1B).

### Differential metabolite analysis

**Figure 2.**
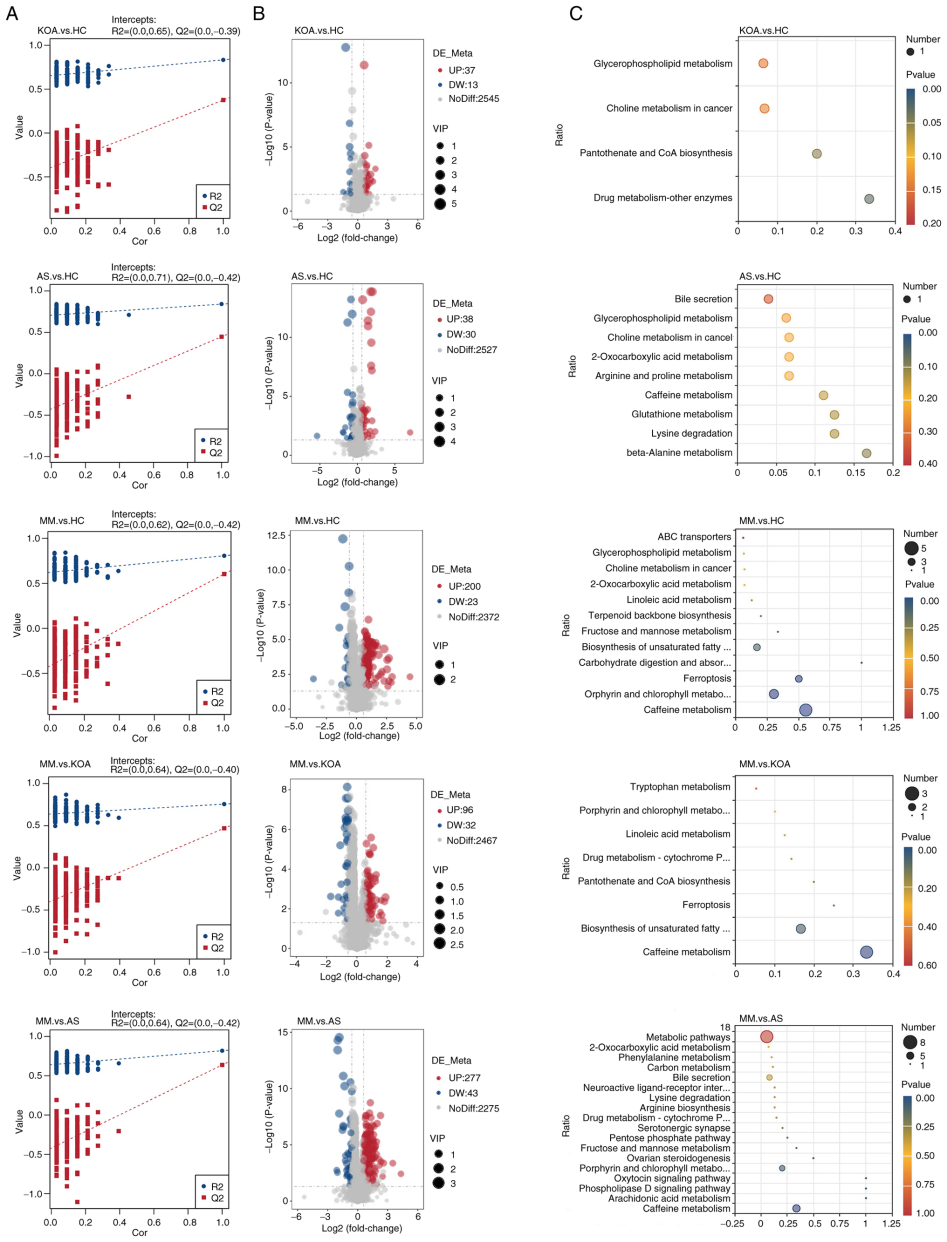
Screening and Functional Analysis of Differential Metabolites. (A) PLS-DA modeling between comparison groups. (B) Volcano plots of differential metabolites identified across comparison groups. (C) KEGG enrichment analysis of differential metabolites among comparison groups. PLS-DA, partial least squares discriminant analysis; KEGG, Kyoto Encyclopedia of Genes and Genomes.

The current study next performed differential metabolite identification. To ensure the reliability of the results, a supervised PLS-DA model was first developed for an improved account for metabolite variation between each comparison group. The PLS-DA model was validated using a permutation test with 200 random numbers. The results showed that all red predictability (Q2) values on the left side were lower than the original points on the right side, R2 and Q2 were close to 1, R2 data were larger than Q2 data and the Q2 regression line had an intercept with the y-axis of <0. This demonstrated that the PLS-DA model was valid and stable (Fig. 2A). Next, volcano plots were constructed for the identification of differential metabolites among each of the five comparison groups and the screening conditions for the identification of differential metabolites were VIP>1, P<0.05 and FC≥2 or FC≤0.5 (Fig. 2B). After obtaining the identification results of differential metabolites among the comparison groups, to determine their functions, KEGG pathway enrichment analysis was performed using the differential metabolites identified in each of the five comparison groups (Fig. 2C). The results showed that the top five pathways in the AS group vs. the HC group comparison were: β-alanine metabolism, lysine degradation, glutathione metabolism, caffeine metabolism and arginine and proline metabolism, all of which were non-significant. In the KOA group vs. the HC group comparison, drug metabolism-other enzymes and pantothenic acid and CoA biosynthesis pathways were markedly enriched. A total of four pathways, caffeine metabolism, porphyrin and chlorophyll metabolism, iron oxidation and carbohydrate digestion and absorption were markedly enriched in the comparison between the MM group and the HC group. The four pathways markedly enriched in the comparison between the MM group and the AS group were caffeine metabolism, arachidonic acid metabolism, phospholipase D signaling pathway and oxytocin signaling pathway. The caffeine metabolism pathway was markedly enriched in the comparison between the MM group and the KOA group.

From the results of the KEGG analysis, it was observed that significant enrichment in caffeine metabolism pathways was observed in the comparison of the MM group with the other three groups (Fig. 2C), suggesting that abnormal caffeine metabolism may influence or promote the development of KOA-AS multimorbidity. The present study further analyzed the caffeine metabolic pathway by comparing its five related metabolites (caffeine, theophylline, 1-methyluric acid, 1-methylxanthine and 1,7-dimethyluric acid) among the HC, KOA, AS and MM groups, to observe the differences between the groups and the overall trend.

**Figure 3.**
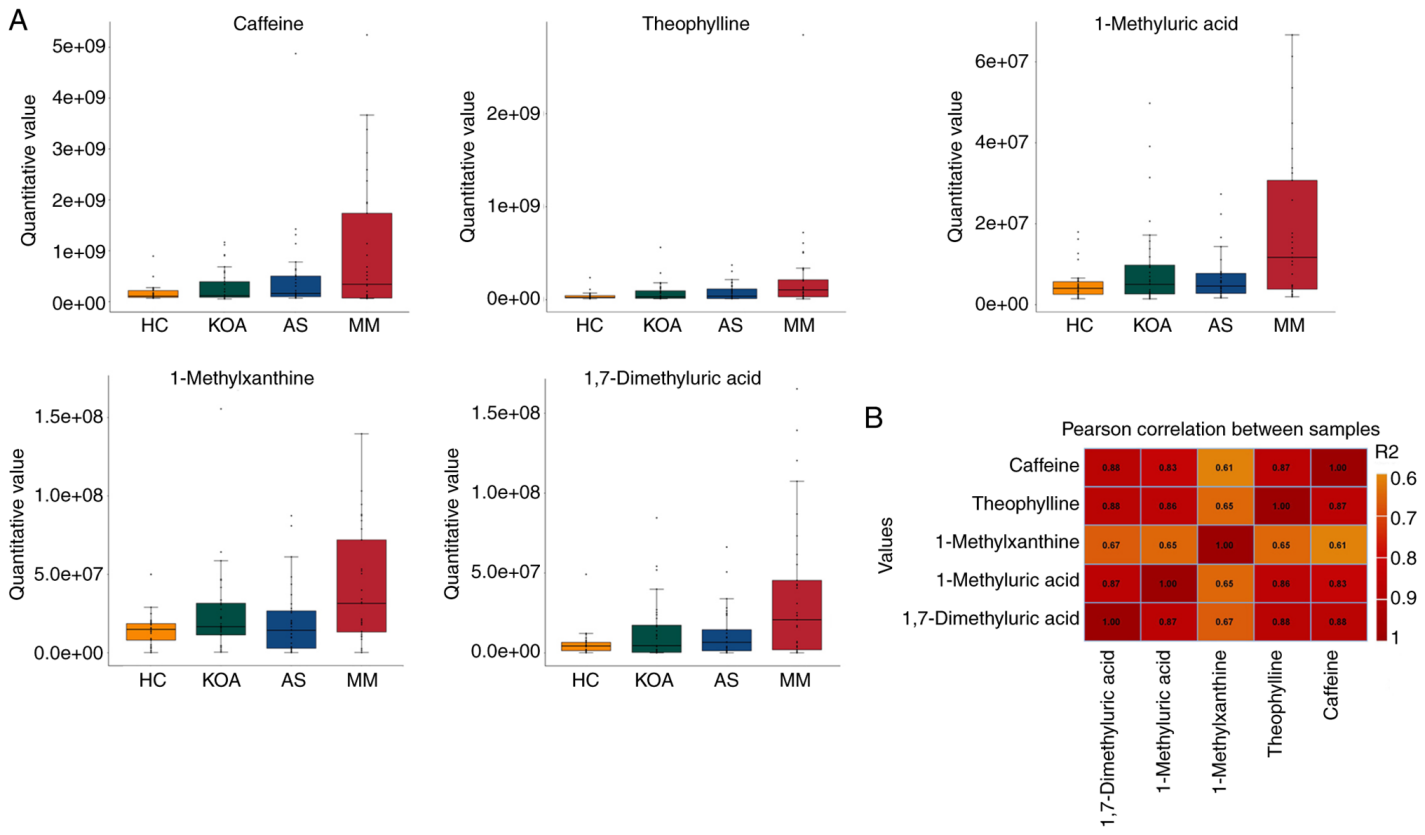
Analysis of Caffeine and Its Metabolites. (A) Box plots for comparison between groups of caffeine and its secondary metabolites. (B) Heat map of Pearson correlation coefficients for relative quantitative values of caffeine and its secondary metabolites.

From the results presented in Table SIV and in the box plots (Fig. 3A), it was indicated that the relative serum levels of the five caffeine metabolism-related metabolites (caffeine, theophylline, 1-methyluric acid, 1-methylxanthine and 1,7-dimethyluric acid) increased progressively with the number of individuals suffering from the disease (healthy control < suffering from mono-disease < suffering from co-disease). It may be suggested that the higher the relative serum levels of caffeine and its secondary metabolites, the higher the risk of developing KOA-AS multimorbidity.

To understand the association between the relative serum levels of these five metabolites, the Pearson correlation coefficients between the relative quantitative values of five metabolites were calculated (Fig. 3B) and it was observed that the correlation coefficients of all five metabolites (r-values) were all >0.6, suggesting that the relative levels of these five metabolites were moderately or highly positively associated with each other.

### Transcriptomics analysis data sources

To further investigate the possible effects of metabolic abnormalities and caffeine on patients with KOA-AS multimorbidity, common DEGs in patients with KOA and patients with AS were assessed using publicly available transcriptomics data to screen hub genes for KOA-AS multimorbidity.

According to the previously established criteria, the GSE48556 dataset for OA (139 samples, 33 controls and 106 patients with OA; mean age, ~ 60 years; the case group suffered from OA in at least two or more sites) and the GSE23746 dataset for AS (95 samples, 19 controls and 76 patients with AS; the case group comprised patients with carotid AS) were obtained. The experimental platform for both datasets was gene expression microarray technology, the specimen types were human peripheral blood mononuclear cells (PBMCs) and the sample sizes were relatively large, which were able to satisfy the analysis needs.

### Screening of common DEGs

**Figure 4.**
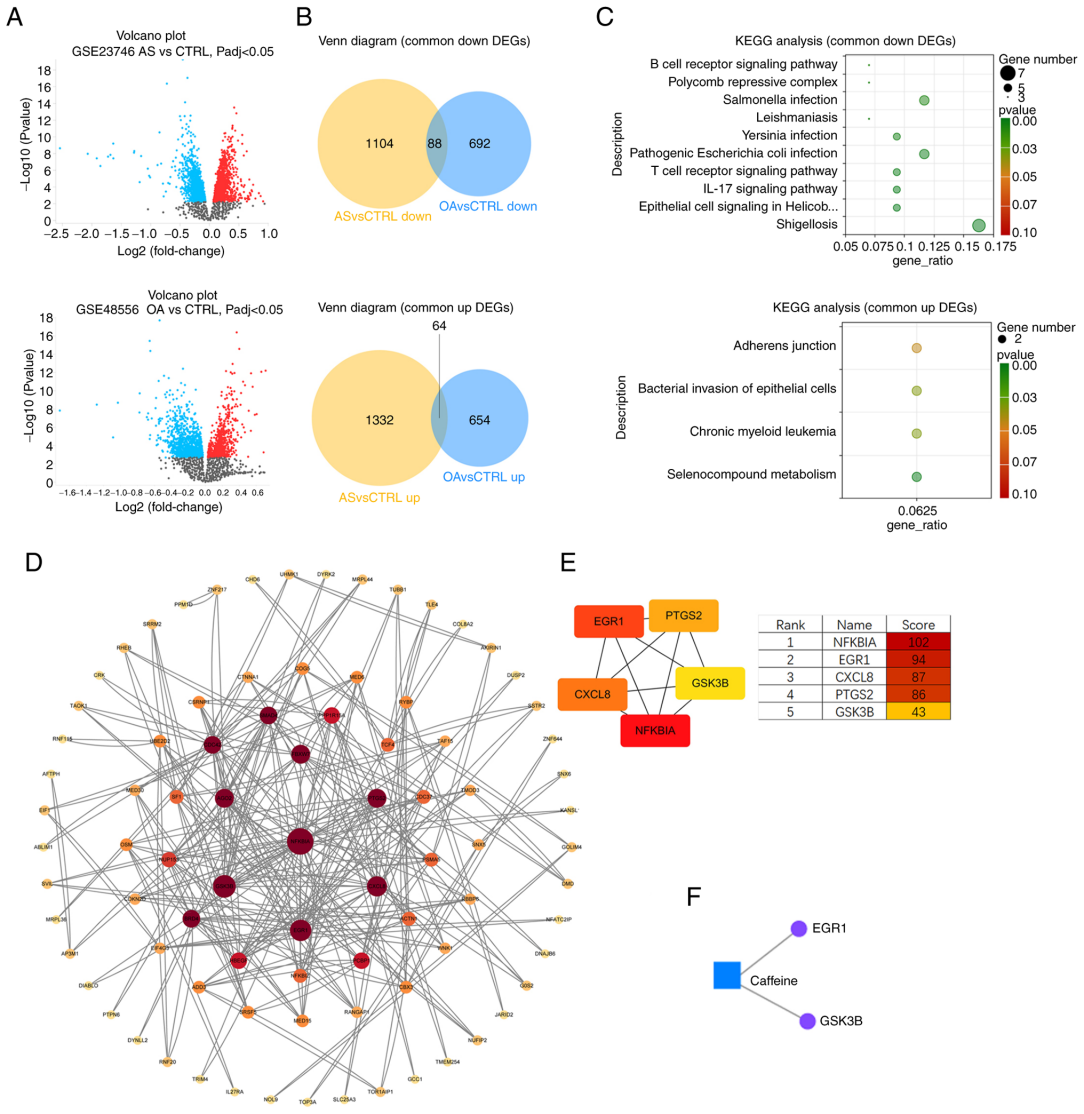
Multi-omics integration and regulatory network analysis based on shared DEGs. (A) Volcano plots of DEGs for the OA dataset and the AS dataset. (B) DEGs shared by OA and AS (categorized into up- and downregulation). (C) KEGG enrichment analysis of shared DEGs. (D) PPI network constructed by DEGs. (E) Top 5 scoring Hub genes. (F) Gene-metabolite interaction network. DEGs, differentially expressed genes; OA, osteoarthritis; AS, atherosclerosis; KEGG, Kyoto Encyclopedia of Genes and Genomes; PPI, protein-protein interaction.

Using GEO2R, an online analysis platform for the GEO database, 2,082 DEGs (including 814 upregulated genes and 1,268 downregulated genes) were obtained from the GSE48556 OA dataset (Fig. 4A) and 2,932 DEGs (including 1,590 upregulated genes and 1,342 downregulated genes) were obtained from the GSE23746 AS dataset. The Venn diagram intersections showed 64 shared upregulated DEGs and 88 shared downregulated DEGs (Fig. 4B).

### KEGG analysis of OA and AS common DEGs

To understand the function of DEGs shared by OA and AS, KEGG functional enrichment analysis was performed on the 64 upregulated DEGs and 88 downregulated DEGs (Fig. 4C). The results showed that upregulated DEGs were enriched in the metabolism of selenium compounds, chronic myeloid leukemia, bacterial invasion of epithelial cells and adhesion junction processes. Downregulated DEGs were mainly enriched in shigellosis, epithelial cell signaling in *Helicobacter pylori* infection, IL-17 signaling pathway, T-cell receptor signaling pathway and pathogenic *E. coli* infection processes.

The enrichment results showed that the common transcriptional features of PBMCs in patients with OA and patients with AS were the disruption (activation or inhibition) of infectious disease-related processes and the suppression of immune system-related pathways.

### PPI analysis for screening hub genes

To understand the interrelationships between the DEGs corresponding to the encoded proteins, all 152 DEGs (64 up- and 88 downregulated) were uploaded to the STRING database to construct the PPI network (Fig. 4D). The PPI network contains a total of 85 nodes and 145 edges. In order to screen the hub genes, the PPI network was imported into Cytoscape software and the cytoHubba plug-in was used to assign values to each protein according to the topological network algorithm. The top five scored genes in the Maximal Clique Centrality mode were analyzed, which were *NFKBIA, EGR1, CXCL8, PTGS2* and *GSK3B* (Fig. 4E).

### Metabolite-gene network analysis

The ‘Network Analysis’ function in Metaboanalyst 6.0 (metaboanalyst.ca/), an online metabolomics analysis platform, was applied to perform Gene-Metabolite Interaction Network Analysis on five caffeine-related metabolites (caffeine, theophylline, 1-methyluric acid, 1-methylxanthine and 1,7-dimethyluric acid) and the top five scoring hub genes (*NFKBIA, EGR1, CXCL8, PTGS2* and *GSK3B*). Caffeine was found to interact with *EGR1* and *GSK3B* (Fig. 4F).

### Validation of clinical samples

**Figure 5.**
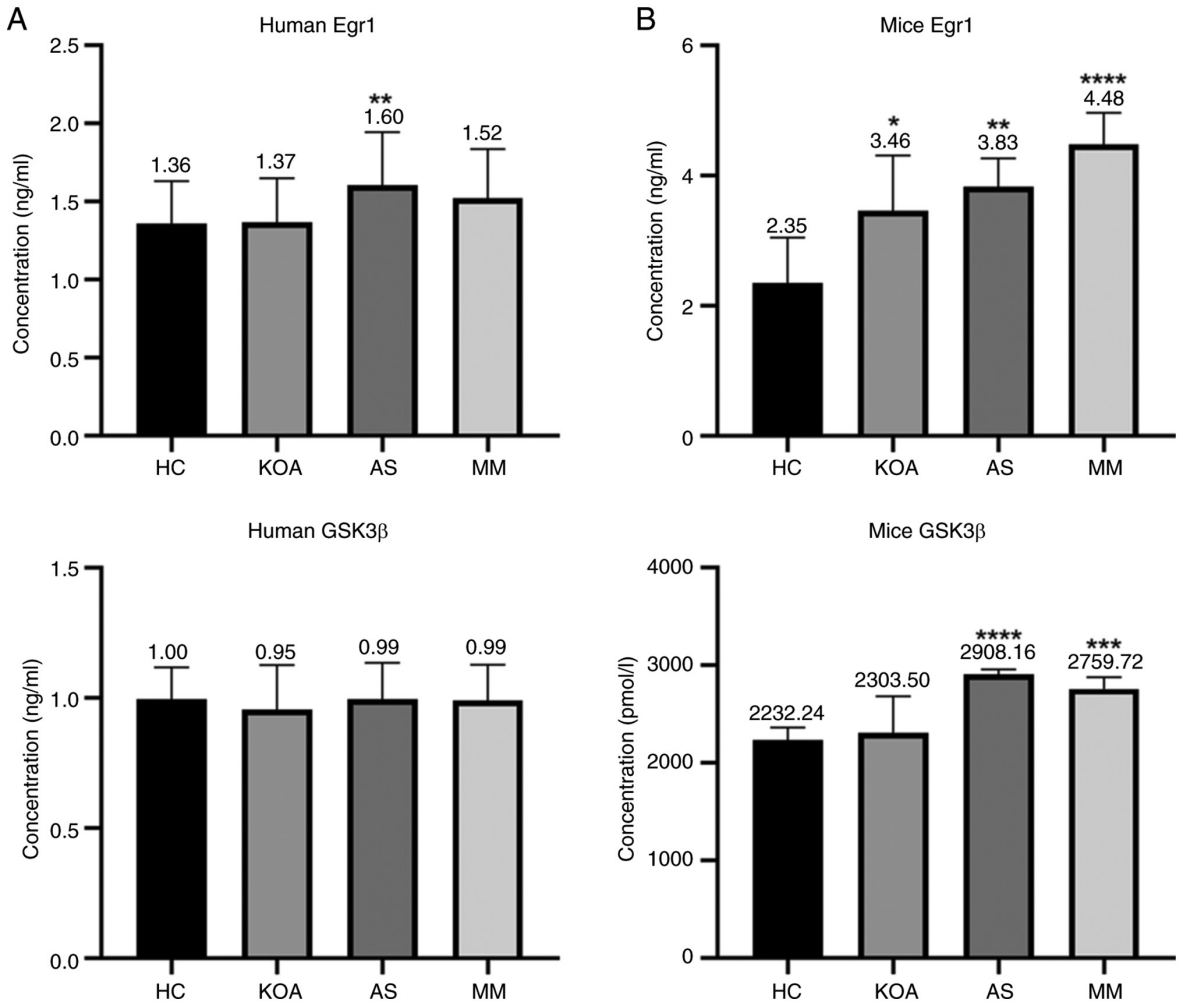
Validation of Serum Egr1 and GSK-3β Protein Levels by ELISA. (A) Relative concentrations of human serum Egr1, GSK-3β proteins in each group. (B) Relative concentrations of mice serum Egr1, GSK-3β proteins in each group. *P<0.05, **P<0.01, ***P<0.0001, ****P<0.00001. Egr1, early growth response 1; GSK-3β, Glycogen Synthase Kinase-3β.

To verify the effect of caffeine on *EGR1* and *GSK3B* genes, the same batch of serum samples used for the metabolomics assay were used and the ELISA method was applied to measure the concentrations of Egr1 and GSK3β proteins in the serum of the aforementioned groups. The standard curves were plotted using CurveExpert 1.4 and the OD450 values were converted to standard units of ng/ml. The correlation coefficients of the standard curves of each batch were r>0.999 and the results were reliable. The bar graph (Fig. 5A) showed that the human serum GSK3β protein concentrations did not differ between groups; whereas the human serum Egr1 protein concentration exhibited an association with AS. Serum Egr1 protein concentration was markedly higher in the AS compared with that in the HC group.

### Validation of disease modeling effects

To validate the hub genes for KOA-AS multimorbidity, a disease model generated in mice corresponding to the clinical study groups was used. After the experimental animals were randomly grouped, they were pre-housed for 1 week and were fed normal chow to acclimate them to the environment before modeling. A total of 1 week after the mice were acclimated to the environment, the mice in the AS and MM groups were fed high-fat chow, whereas the KOA model was surgically constructed by applying the modified Hulth method to the mice in the KOA and MM groups from week 3.

**Figure 6.**
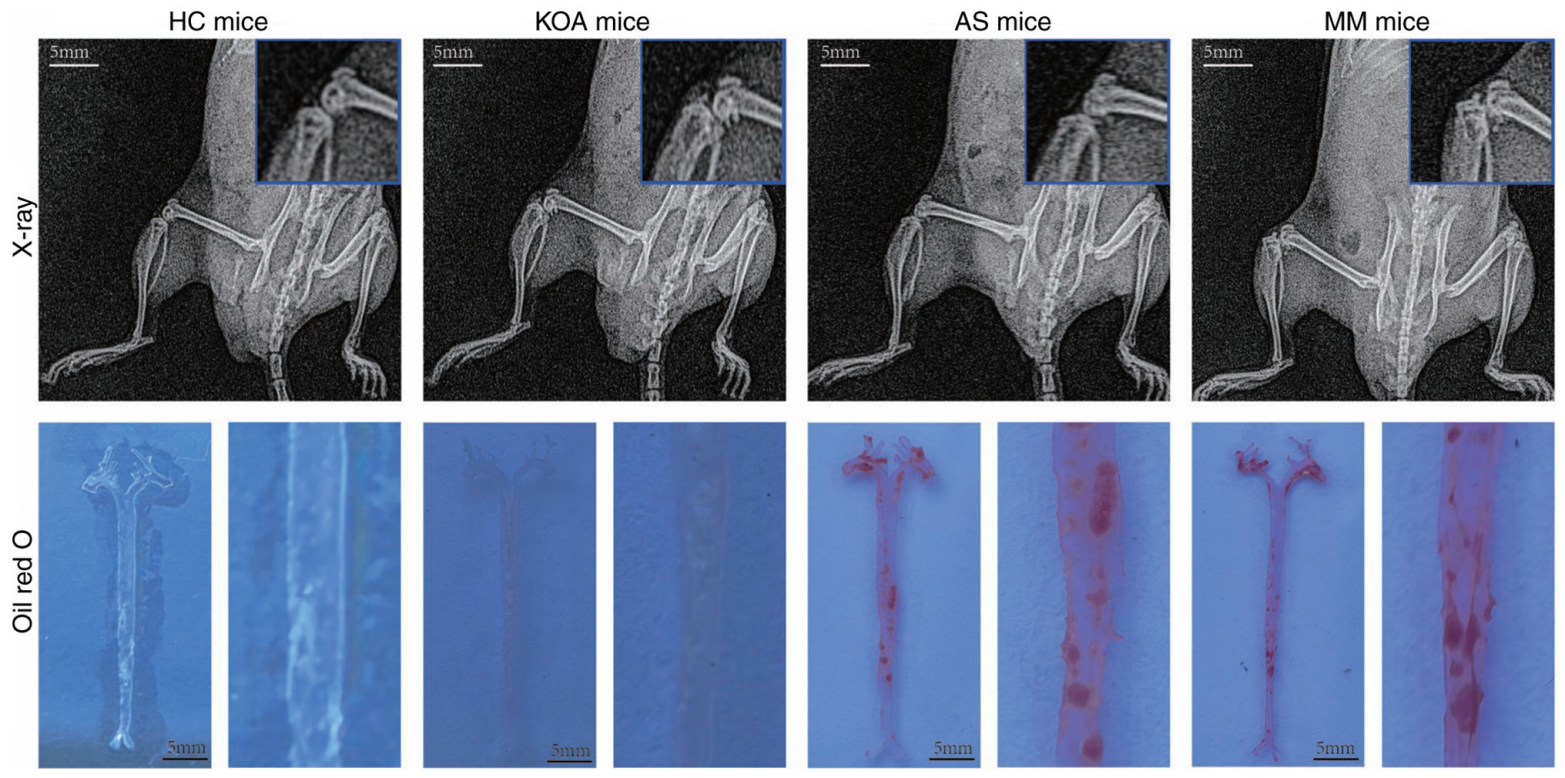
Results of animal model construction for each group of diseases.

The modeling effect of the mice in each group was verified after 12 weeks of high-fat chow feeding and the materials (Aorta, cartilage, blood) were taken at the same time (Fig. 6). X-ray examination showed that the knee joints of mice in the KOA and MM groups had obvious osteoclasts, whereas the knee joints of mice in the other two groups were smooth and free of osteoclasts. The results of Oil Red O staining showed that lipid plaques had formed in the aorta of mice in the AS and MM groups, whereas no lipid plaques were observed in the other two groups. Based on these results, it was determined that the animal disease models were successfully constructed.

### Differential expression of the hub genes in the serum of animal disease models

The concentrations of two proteins, Egr1 and GSK3β, in the serum of each group of mice were determined by ELISA. The standard curve was plotted using CurveExpert 1.4 and the OD450 values were converted to ng/ml or pmol/l. The standard curves for each batch showed correlation coefficients r>0.999, confirming reliability (Fig. 5B).

The serum Egr1 protein concentration of mice was associated with both KOA and AS and there were significant differences in the levels in the KOA, AS and MM group compared with those in the HC group. In addition, there was a rising trend with the increase in the number of diseased mice (healthy control < suffering from a single disease < suffering from a co-morbid disease). The results were in agreement with the metabolite-gene network analysis, thus indicating that *EGR1* may be the key gene for KOA-AS multimorbidity.

Serum GSK3β protein concentration in mice was associated with AS only, with significant differences in the AS and the MM group compared with the HC group.

### Differential expression of hub genes in the cartilage tissues of animal disease models

**Figure 7.**
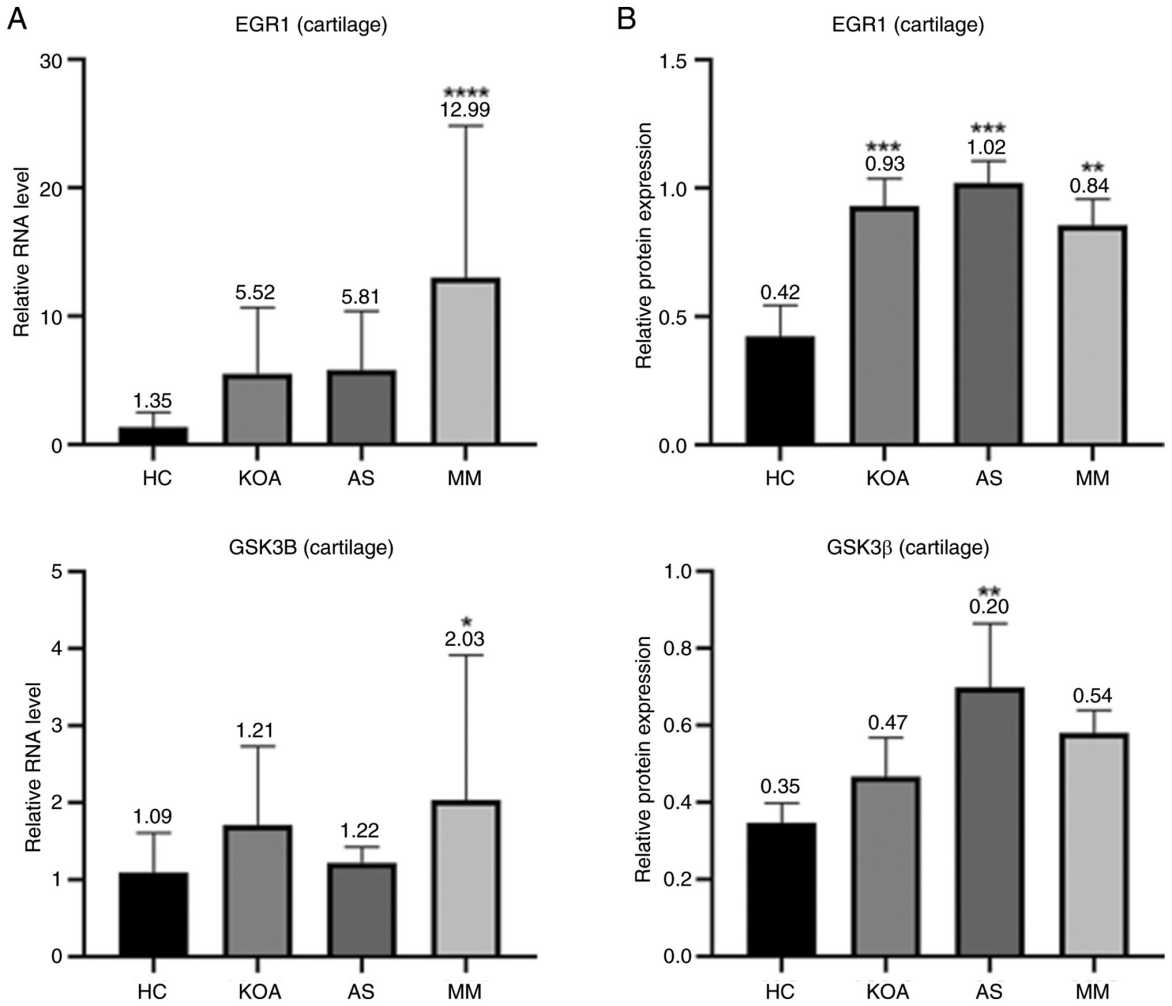
Molecular validation of *EGR1* and *GSK3B* Expression in Cartilage Tissue. (A) Relative RNA levels of *EGR1* and *GSK3B* in mouse cartilage in each group. (B) Protein expression levels and electropherograms of cartilage Egr1 and GSK-3β in mice of each group. *P<0.05, **P<0.01, ***P<0.0001, ****P<0.00001.Egr1, Early Growth Response 1; GSK-3β, glycogen synthase kinase-3β.

The bar graphs (Fig. 7) showed that in mouse articular cartilage tissues, the RNA transcript levels of *EGR1* and the protein expression levels of Egr1 were markedly higher in the KOA, AS and MM group than those in the HC group, which was in line with the results shown in the mouse serum samples. The results regarding GSK3β did not show a uniform pattern and the expression levels of GSK3β in mouse cartilage tissues only showed an association with AS, which was consistent with the results in the mouse serum; however, the RNA transcript levels of *GSK3B* in cartilage tissues were only associated with KOA. In conclusion, Egr1 exhibited a high association with KOA-AS multimorbidity in mouse serum, which was further verified in the assessment of mouse cartilage tissues and its corresponding gene *EGR1* is expected to be a common therapeutic target for KOA and AS.

### ROC curve analysis of metabolites

**Figure 8.**
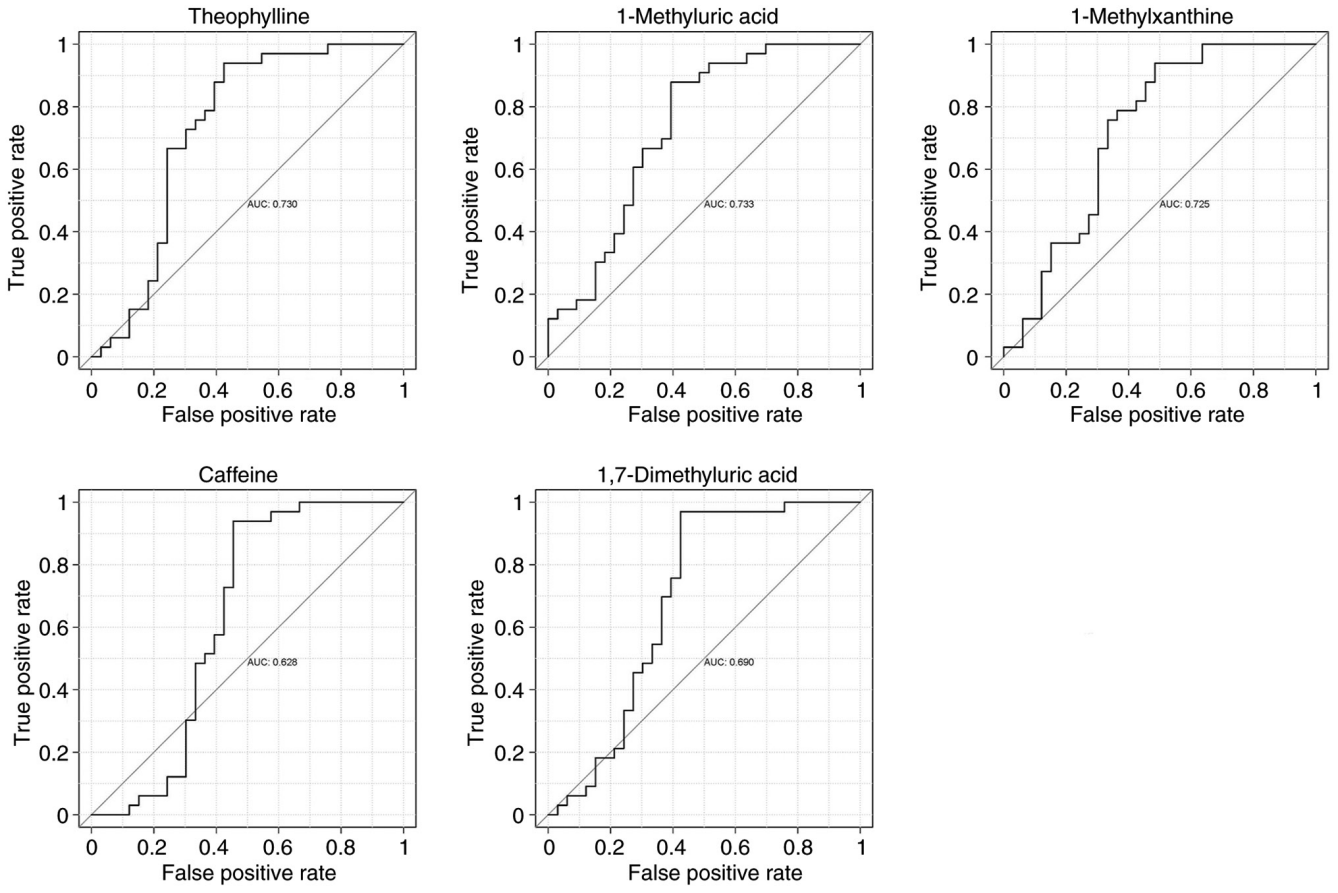
ROC curve analysis of caffeine and its secondary metabolites. ROC, receiver operating characteristic.

To assess the diagnostic value of caffeine and its secondary metabolites (theophylline, 1-methyluric acid, 1-methylxanthine and 1,7-dimethyluric acid), a subject workup characterization (ROC curve analysis) was performed using relative quantitative values of metabolites from metabolomics analysis in the HC and MM groups (Fig. 8), which showed that theophylline, 1-methyluric acid and 1-methylxanthine had a better potential to become diagnostic markers (AUC >0.7), whereas caffeine and 1,7-dimethyluric acid had a relatively average performance (AUC >0.6).

### Predicting KOA-AS multimorbidity drugs

*EGR1* was imported into the DGidb database for drug prediction and a total of two small-molecule targeted drugs (GENIPIN, BRIVOLIGIDE) were exported; the DGidb database online scoring software was used to calculate the interaction scores of the drugs with the genes (Table II). The results revealed that the interaction scores of these two drugs were high and both of them were not approved for the market, thus indicating that *EGR1* may be a potential future drug target.

## Discussion

From the point of view of metabolic abnormalities, in the present study population, obesity (BMI), blood glucose, a previous history of hypertension and a previous history of diabetes mellitus were associated with both KOA and AS, whereas systolic blood pressure, diastolic blood pressure and triglycerides were mainly associated with AS. These findings suggested that metabolic abnormalities may be more pronounced in those with AS than those with KOA in a population with a mean age of 40 years. Consistent results were also obtained in the intergroup clustering heat map analysis of serum metabolomics, where the serum metabolic profile of the KOA group was closer to that of the HC group among the two mono-disease groups, whereas that of the AS group was closer to that of the MM group, suggesting that metabolic abnormalities have a greater effect on AS. In addition, the results of the analysis of blood pressure (associated only with AS) and previous history of hypertension (associated with both KOA and AS) were not consistent in the same population, presumably influenced by the control of antihypertensive medication.

The present results suggested that OA and AS are interrelated through the metabolic syndrome, with various components of the metabolic syndrome (obesity, diabetes mellitus, dyslipidemia and hypertension) associated with OA (39) and it has been suggested that OA is a phenotype of the metabolic syndrome (40). In addition, metabolic syndrome is a risk factor for CVDs, which involves a variety of pathologies such as dyslipidemia, elevated blood glucose and elevated blood pressure, all of which increase the risk of AS (41).

The mean age of the present study population was low, which may explain the lack of abnormalities in metabolic pathways associated with metabolic syndrome in the results of metabolomics analysis. On the other hand, however, conducting the present study in a relatively young population may help to understand the earliest onset state of the disease. Although not markedly enriched for abnormalities in pathways associated with metabolic syndrome, caffeine metabolism was observed to be markedly enriched in all three comparison groups based on the results of KEGG enrichment analysis, suggesting that abnormalities in caffeine metabolism may be associated with KOA-AS multimorbidity. In the single-indicator analysis for caffeine and its secondary metabolites (theophylline, 1-methyluric acid, 1-methylxanthine and 1,7-dimethyluric acid), a consistent trend was observed in which the levels of these metabolites were markedly increased with the number of diseased individuals in the study population. The higher the relative serum levels of caffeine and its secondary metabolites in the current study population, the higher the risk of developing KOA-AS multimorbidity and it could be hypothesized that caffeine metabolism capacity varies between individuals and that caffeine metabolism disorders may exist. It is hypothesized that caffeine metabolism impairment results in progressive serum caffeine accumulation. Consequently, elevated caffeine levels drive inflammatory disease pathogenesis through *EGR1* gene dysregulation and the induction of a pro-inflammatory milieu. Previous studies have reported that the CYP1A2 gene rs762551 polymorphism is associated with individual caffeine metabolism capacity (42,43). Individuals with the AA genotype have been identified as ‘slow metabolizers’ of caffeine, exhibiting markedly lower caffeine clearance rates compared with AC/CC genotype ‘fast metabolizers’ (42,43). The inferred ‘caffeine metabolism disorder’ in our study may be related to this SNP, indicating that differences in serum caffeine levels are influenced by both lifestyle habits and genetic factors. The current study did not include genotyping of this SNP locus nor collected data on caffeine intake. This will be a key direction for future research.

Previous studies on the relationship between caffeine and health are controversial; a previous study suggesting that caffeine intake is protective against CVDs and that long-term caffeine intake develops tolerance (44), whereas others have demonstrated that caffeine intake raises adrenaline levels and blood pressure in the short term (45), which may have an acute detrimental effect on aortic stiffness in patients with hypertension (46). This suggests that elevated blood pressure may be the earliest in the spectrum of metabolic abnormalities associated with KOA-AS multimorbidity.

Bioinformatics analysis using public transcriptomics datasets showed that the common feature of OA and AS in the high age group (mean age >60 years) was inflammation, which is consistent with the expectation of the present study, as evidenced by the disruption of infectious disease-related pathways and the suppression of some immune system signaling pathways. The five key genes screened in the current study (*NFKBIA, EGR1, CXCL8, PTGS2* and *GSK3B*) are also involved in inflammatory responses to varying degrees. Combining the present findings with those of previous studies, it may be extrapolated that the inflammatory state in the late stage of the disease in patients with KOA-AS multimorbidity is caused by numerous metabolic abnormalities in the early stage.

In the metabolite-gene interaction network analysis, it was revealed that *EGR1* and *GSK3B* interacted with caffeine and through further review of the literature, it was demonstrated that caffeine may have inconsistent effects on *EGR1* and *GSK3B* under different circumstances. Previous studies suggested that caffeine activates GSK3β by inhibiting its phosphorylation (47,48); as for *EGR1*, some studies have shown that *EGR1* expression is downregulated in hepatocytes after treatment with a certain concentration of caffeine (49); and that caffeine upregulates SIRT3 expression by inhibiting the *EGR1* signaling pathway in astrocytes of the central nervous system in mice (50). Others have shown that caffeine has a promoting effect on *EGR1* expression, such as in mice treated with caffeine combined with cocaine, where the prefrontal cortex *EGR1* expression was shown to be upregulated (51). This suggests that caffeine has varying effects on *EGR1* in different tissues or physiological states.

Based on the findings that *EGR1*/Egr1 was markedly upregulated in both serum and cartilage tissues (RNA/protein levels) in the KOA, AS and MM groups in advanced disease models, whereas in the serum of clinically mildly ill patients, Egr1 was elevated only in the AS group, its reliability may be suggested as a KOA-AS multimorbid hub gene with transformation into a generalized marker in advanced stages. By contrast, the regulation of GSK3β showed complexity; its protein levels (serum and cartilage) were elevated in the AS and MM groups, whereas its RNA levels in cartilage were associated with KOA, suggesting post-transcriptional regulation or functional differentiation. Notably, disease stage markedly affected the results, with widespread abnormalities of Egr1/GSK3β in late models (compared with early clinical samples) supporting the inference that late inflammation is triggered by early metabolic abnormalities. In addition, diseased mice without caffeine intake still exhibited elevated serum Egr1 and GSK3β, suggesting that their levels are regulated by factors other than caffeine.

In previous studies, *EGR1* has been reported as a possible hub gene of KOA (52) and AS (53). To the best of the authors' knowledge, the present study is the first to propose the key role of *EGR1* in KOA-AS multimorbidity and to construct an innovative disease model of KOA-AS multimorbidity. Notably, the results of the metabolite-gene network analysis were verified by mice experiments. *EGR1* has also been reported to be associated with metabolic disorders such as metabolic dysfunctions, insulin resistance and others (54,55), which supports the association between metabolic abnormalities and *EGR1.* As a transcriptional regulator, *EGR1* plays a key role in the regulation of macrophage inflammatory response and it may be hypothesized that metabolic abnormalities could promote inflammation by regulating the expression of *EGR1*.

In the clinical application analysis, the present study identified three caffeine secondary metabolites (theophylline, 1-methyluric acid and 1-methylxanthine) as having potential to become diagnostic biomarkers and *EGR1* was considered a drug target with some value. Although GENIPIN (a drug predicted by *EGR1*), has not been approved for marketing, experimental studies have suggested that hydrogels linked by GENIPIN can reduce joint wear and tear in patients with OA (56) and GENIPIN can be used to make scaffolds for cardiovascular use (57). In addition, it has been suggested that GENIPIN may ameliorate the effects of hyperlipidemia and lipid accumulation in the liver (58). These findings also corroborate the reliability of the predicted results.

In conclusion, the present study not only verified previous studies on KOA-AS multimorbidity, but also explored the association between KOA and AS and the possible formation mechanisms of KOA-AS multimorbidity from numerous perspectives. Abnormal caffeine metabolism was revealed to be an early risk factor for KOA-AS multimorbidity and potential hub genes for KOA-AS multimorbidity were identified. Preliminary predictions on the clinical value of these key molecules for KOA-AS multimorbidity were conducted, which are expected to provide new directions for diagnostic and therapeutic studies of KOA-AS multimorbidity. Finally, the present study has the following shortcomings: i) The available information could not determine the reasons for the differences in serum caffeine levels between the groups and caffeine intake needs to be assessed to confirm this; ii) the current findings do not clarify the specific effects of caffeine and the location of its effects on KOA-AS multimorbidity and interventional experiments need to be performed to further demonstrate this; and iii) predictions about the value of clinical applications need to be verified by further experiments.

## Supplementary Material

Supporting Data

## Figures and Tables

**Table I.** Clinical indicators and KOA-AS multimorbidity.

	Clusters			
	
Clinical indicators	HC (n=43)	KOA (n=43)	AS (n=43)	MM (n=43)
BMI	24.15±3.33	26.15±2.94^a^	25.69±3.53^b^	27.13±3.44^c^
Triglycerides	1.4±0.98	1.42±0.8	2.24±1.58^a^	2.36±3.21
Total cholesterol	4.77±1.03	4.96±0.76	5.03±1	5.15±1.48
Fasting glucose	5.16±0.41	5.48±0.7^b^	5.63±1.12^b^	6.73±2.09^cde^
Systolic blood pressure	113.86±13.25	118.51±12.41	131.86±13.97^c^	135.42±11.29^ce^
Diastolic blood pressure	71.09±7.76	73.51±9.29	83.86±10.14^c^	85.44±9.19^ce^
Past medical history				
Hypertension	2/43	7/43	9/43^b^	24/43^cef^
Hyperlipidemia	1/43	6/43^b^	7/43^b^	2/43
Diabetes mellitus	1/43	4/43	1/43	13/43^cfg^
Coronary heart disease	0/43	0/43	0/43	3/43
Other	3/43	1/43	0/43	4/43^h^
Total	7/43	14/43	16/43^b^	33/43^cef^

KOA, knee osteoarthritis; AS, atherosclerosis; HC, healthy control group; MM, KOA-AS multimorbidity group; BMI, Body Mass Index.

a P<0.01,

b P<0.05;

c P<0.001 vs. HC;

d P<0.01,

f P<0.001,

h P<0.05 vs. AS;

e P<0.001,

g P<0.05 vs. KOA group

**Table II.** DGidb Database output.

Gene	Drug	Regulatory approval	Interaction Score
*EGR1*	GENIPIN	Not approved	52.203799
*EGR1*	BRIVOLIGIDE	Not approved	26.101899

EGR1, Early Growth Response 1.

## Data Availability

The data generated in the present study may be requested from the corresponding author.

## Authors' information

Dr Zhang Keshi ORCID: 0000-0003-3547-1964. Dr Zhenpeng Guan ORCID: 0000-0002-4487-7918.
