# Supplementary material for: Identification of multimorbidity hub genes for knee osteoarthritis-atherosclerosis and potential clinical applications
Source: Mol Med Rep. 2025 Nov 12;33(1):40. doi: 10.3892/mmr.2025.13750 (PMC12641210; doi:10.3892/mmr.2025.13750)
Supplement: Supporting Data [file Supplementary_Data.pdf]

Table SI. Primer sequences.

Gene	Primer sequence, 5'→3'
GSK3B	F: TACCACTCAAGAACTGTCAA<br>R: CACGGTCTCCAGCATTAG
EGR1	F: GGCTCTTAATACCACCTACC<br>R: TCACTACGACTGAAGTTACG
GAPDH	F: AGAAGGTGGTGAAGCAGGCATCT<br>R: CGGCATCGAAGGTGGAAGAGTG

GSK3B, Glycogen Synthase Kinase-3β; EGR1, early growth response 1; F, forward; R, reverse.

Table SII. Subgroups and basic characteristics.

Characteristic	HC (n=43)	KOA (n=43)	AS (n=43)	MM (n=43)	Total (n=172)
Mean age, years	37.14±3.59	43.30±8.32	37.91±4.63	51.19±5.59	42.38±8.07
Median age, years	36	41	38	51	40
Age range	31-46	33-59	27-49	36-59	27-59
Male (%)	26 (60.47)	30 (69.77)	28 (65.12)	39 (90.70)	123 (71.51)
Female (%)	17 (39.53)	13 (30.23)	15 (34.88)	4 (9.30)	49 (28.49)

KOA, knee osteoarthritis; AS, atherosclerosis; HC, healthy control group; MM, KOA-AS multimorbidity group.

Table SIII. Subgroups and basic characteristics for metabolomics.

Characteristics	HC (n=33)	KOA (n=33)	AS (n=33)	MM (n=33)	Total (n=132)
Mean age, years	36.24±3.10	42.21±9.13	37.45±3.65	49.91±7.28	41.45±8.28
Median Age	35	38	38	51	38
Age range	31~45	33~59	30~44	34~59	30~59
Male (%)	19 (57.60)	22 (66.70)	19 (57.60)	29 (87.90)	89 (67.42)
Female (%)	14 (42.40)	11 (33.30)	14 (42.40)	4 (12.10)	43 (32.58)

KOA, knee osteoarthritis; AS, atherosclerosis; HC, healthy control group; MM, KOA-AS multimorbidity group.

Table SIV. Relative serum levels of caffeine and its secondary metabolites.

**Metabolite**	**HC**	**KOA**	**AS**	**MM**
Caffeine	1692.68	2997.53	4759.91	9840.81
Theophylline	380.01	783.62	803.83	2448.50
1-Methyluric acid	50.15	91.97	66.89	179.43
1-Methylxanthine	148.38	292.02	200.16	412.05
1,7-Dimethyluric acid	54.80	136.77	120.97	354.09

Data are presented as relative levels scaled by a factor of 10^{-4}. KOA, knee osteoarthritis; AS, atherosclerosis; HC, healthy control group; MM, KOA-AS multimorbidity.
